# Genetic Background of Patients from a University Medical Center in Manhattan: Implications for Personalized Medicine

**DOI:** 10.1371/journal.pone.0019166

**Published:** 2011-05-04

**Authors:** Bamidele O. Tayo, Marie Teil, Liping Tong, Huaizhen Qin, Gregory Khitrov, Weijia Zhang, Quinbin Song, Omri Gottesman, Xiaofeng Zhu, Alexandre C. Pereira, Richard S. Cooper, Erwin P. Bottinger

**Affiliations:** 1 Department of Preventive Medicine and Epidemiology, Loyola University Chicago Stritch School of Medicine, Maywood, Illinois, United States of America; 2 Charles R. Bronfman Institute for Personalized Medicine, Mount Sinai School of Medicine, New York, New York, United States of America; 3 Department of Biostatistics and Epidemiology, Case Western University, Cleveland, Ohio, United States of America; 4 University of Sao Paulo Medical School, Sao Paulo, Brazil; Ohio State University Medical Center, United States of America

## Abstract

**Background:**

The rapid progress currently being made in genomic science has created interest in potential clinical applications; however, formal translational research has been limited thus far. Studies of population genetics have demonstrated substantial variation in allele frequencies and haplotype structure at loci of medical relevance and the genetic background of patient cohorts may often be complex.

**Methods and Findings:**

To describe the heterogeneity in an unselected clinical sample we used the Affymetrix 6.0 gene array chip to genotype self-identified European Americans (N = 326), African Americans (N = 324) and Hispanics (N = 327) from the medical practice of Mount Sinai Medical Center in Manhattan, NY. Additional data from US minority groups and Brazil were used for external comparison. Substantial variation in ancestral origin was observed for both African Americans and Hispanics; data from the latter group overlapped with both Mexican Americans and Brazilians in the external data sets. A pooled analysis of the African Americans and Hispanics from NY demonstrated a broad continuum of ancestral origin making classification by race/ethnicity uninformative. Selected loci harboring variants associated with medical traits and drug response confirmed substantial within- and between-group heterogeneity.

**Conclusion:**

As a consequence of these complementary levels of heterogeneity group labels offered no guidance at the individual level. These findings demonstrate the complexity involved in clinical translation of the results from genome-wide association studies and suggest that in the genomic era conventional racial/ethnic labels are of little value.

## Introduction

With the dramatic decline in the cost of sequencing and the availability of well annotated databases genomic research is moving rapidly toward the clinical arena [1], [2], [3]. Risk loci have been identified for many common diseases and while they collectively explain a small proportion of the heritable risk modest effects have been noted for markers associated with conditions such as focal segemental glomerular sclerosis, hyperlipidemia, Crohn's disease, adult macular degeneration, type 2 diabetes, rheumatoid arthritis, schizophrenia and bipolar disorder, and coronary artery disease among others [4], [5], [6], [7], [8], [9], [10], [11], [12]. Of more immediate clinical relevance has been the discovery of genetic variants which influence the action of pharmacologic agents [13]. Because these loci are unlikely to have been under selective pressure, variants altering drug metabolism have in some cases increased to reasonable frequency and can be associated with large effects [14]. As more geographic populations are studied with high density genotype arrays it is also becoming apparent that allele frequencies for the relevant markers can vary widely [5], [15], [16], [17].

These emerging data must be incorporated into a strategy that positions genomic medicine for a clinical role. Because virtually all risk loci have been identified via proxy markers, it cannot be assumed that the haplotypes that are being identified in the populations where the original findings are made will carry the causal mutations in other geographically separated groups. For example, a recent analysis of the three HapMap populations showed considerable heterogeneity of allele frequencies for loci associated with 26 common diseases [16]. Likewise, the fat mass and obesity associated (*FTO*) locus, which has the strongest known association with obesity in Europeans, shows a complex and inconsistent pattern in African-origin populations [15], [17]. The generalizability of the current generation of published risk markers in all racial/ethnic groups cannot therefore be taken for granted.

In the initial phase much of the interest in molecular studies of population differentiation was focused on large continental groupings [18], [19], [20]. Fine-scale population structure has now been examined using dense genotype data in Europe and North America, and somewhat more limited data has become available from Latin America, Africa and Asia [18], [19], [21], [22], [23]. Most of these studies have attempted to identify “source” populations and describe migration and other demographic patterns over a historical framework [21], [23], [24]. In a sense, therefore, this research has been “backward looking”, as formalized in the Human Diversity Project [25]. However migration and gene flow between populations that had historically been geographically distant has accelerated in the modern era and many large metropolitan areas are now exceedingly diverse. Considered as a “city region”, New York City had an estimated population of 20 million; in 2005 36% of the residents of New York City proper were foreign born, speaking 170–200 languages [26], [27]. In this cosmopolitan setting the standard US racial/ethnic categories – ie, black, white, Hispanic, Asian – become particularly problematic.

Mount Sinai Medical Center serves a diverse community in northern Manhattan with outpatient visits totaling 800,000/year. The Charles R. Bronfman Institute for Personalized Medicine at Mount Sinai has initiated a program of research aimed at translating the growing body of evidence on genetic susceptibility for chronic disease and drug responsiveness into clinical practice. As an initial step we collected dense genotype data on a sample of 977 outpatients served by our institution who self-identified into 3 major racial/ethnic groups. Genotype array analysis was used to assess patterns of gene flow between the groups and consistency and distribution of haplotypes at a series of loci known to predispose to common disease or influence the metabolism of drugs.

## Methods

### Ethics statement

This research study was reviewed and approved by the ethics review board of the Program for the Protection of Human Subjects (PPHS) of Mount Sinai School of Medicine under project # HSD09-00030. The Mount Sinai Biobank Project (IRB # 07-0529 0001 02 ME) is an IRB-approved research protocol with IRB-approved informed consent forms. All study participants provided written informed consent.

### Participant recruitment

Study participants were recruited from the Biobank Program of the Institute of Personalized Medicine at Mount Sinai Medical Center. The primary sample consisted of 1030 self-identified African Americans, European Americans or Hispanics. The majority of the Hispanic participants were from the Caribbean, primarily the Dominican Republic and Puerto Rico. One subject from each group and one CEPH trio family were replicated for the purpose of quality control of genotype data. The project was reviewed and approved by the Institutional Review Boards of both Mount Sinai Medical Center and Loyola University Chicago Stritch School of Medicine.

### Genotyping and quality assessment

Genotyping was carried out on genomic DNA from 1030 subjects using the Affymetrix 6.0 gene chip. Genotyping was performed in batches and within each batch samples were randomized with respect to race/ethnicity, gender and diagnostic status. Selected samples from each race plus a CEPH trio sample were also replicated in each batch for the purpose of assessing batch effect on genotypes. Quality control procedures were performed with the Whole Genome Analysis software (Golden Helix) and PLINK [28] (http://pngu.mgh.harvard.edu/purcell/plink/). The chip analysis provided data on 909,600 SNPs of which 905,384 mapped to the dbSNP rsID. Of the 1030 samples, those that broadly failed genotyping (n = 36), had gender inconsistency (n = 5) or had missing genotype proportion >0.05 (n = 7) were excluded. Similarly, 60,869 SNPs with missing genotype rate >5% and 10,889 SNPs with MAF<0.01 were excluded. Using the data on batch-replicated samples, mean genotype concordance rate between batches was estimated to be 99.65±0.08%. Investigation of batch effect on genotypes revealed 1,236 SNPs with substantial deviations associated with batch effect and these were dropped. In addition, SNPs found to have significantly differential missing rates (n = 217) between patients with specific diagnoses and those failing Hardy-Weinberg equilibrium (HWE) test (*p-value = 0.001*) (n = 2,587) were also excluded. We estimated inbreeding coefficients and genome-wide identity-by-descent (IBD) sharing among pairs of samples using the software PLINK [28]. Finally, one additional sample with an inbreeding coefficient greater than four standard deviations of the mean coefficient was dropped. There was no significant evidence of excess sharing of IBD proportion either due to sample contamination, duplication, or cryptic relatedness. The final quality-controlled cleaned dataset thus consisted of 977 unrelated adult subjects – 324 African Americans, 326 European Americans and 327 Hispanics with genome-wide information on 829,586 SNPs.

### Creation of marker sets for population structure analysis

For population structure analysis the cleaned dataset was merged with datasets from the International HapMap Project [29] and prior studies conducted by the Department of Preventive Medicine at Loyola. The HapMap data consisted of samples of African ancestry in the southwest USA (ASW) (n = 71) and of Mexican ancestry in Los Angeles, California (MEX) (n = 71). Descriptions of the sample, genotyping and quality control of the genotype data have been provided elsewhere [29], [30]. The Loyola dataset consisted of a population-based Yoruba sample from Nigeria (YOR) (n = 334), African Americans from Maywood, IL (AMW) (n = 204), and Brazilians (BRZ) (n = 109). The YOR and AMW samples were recruited as controls in a study of genetics of hypertension [31], [32]. Data from appropriate Native American groups were not available. The genotype data were generated on Affymetrix 6.0 chip and details of the samples, genotyping and quality control procedures have been described elsewhere [31]. There were 1770 samples in the combined dataset with genotypes on 599,857 SNPs. The sample genotyping rate in the combined dataset was >0.99 and the MAF of every SNP in each of the subpopulations was at least 0.01.

Two different marker sets were created from the combined dataset for population structure analysis. These markers were chosen to ensure that the SNPs were not in strong linkage disequilibrium (LD) and to make the analysis computationally efficient. For the first marker set, we excluded SNPs with missing genotype rate >0.1%, then used the software PLINK [28] to prune the remaining 238,533 SNPs using pairwise linkage disequilibrium 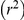 maximum threshold of 0.2 in 50 SNP widows, shifting and recalculating every 5 SNPs. The resulting subset of SNPs consisted of 100,133 SNPs distributed across the genome. The second marker set was created based on average genetic distance difference 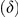 between African and European ancestral populations. Using the HapMap allele frequencies, data for samples of Yoruba in Ibadan, Nigeria (YRI) and CEPH (Utah residents with ancestry from northern and western Europe) (CEU), 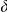 was computed as the sum of the absolute differences between the allele frequencies [33] in the two samples. SNPs with 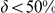 were then excluded from the combined dataset and the remaining 67,124 SNPs were pruned using 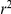 maximum threshold of 0.2 in 50 SNP widows, shifting and recalculating every 5 SNPs. The resulting subset consisted of 28,783 SNPs spread across the genome. The two marker sets were subsequently used separately for population structure analysis.

### Principal component and multidimensional scaling analysis

Principal component analysis was performed with the Whole Genome Analysis software (Golden Helix). The first two components had large eigenvalues compared to the remaining components. These two components were therefore extracted and used as covariates to adjust for stratification in the candidate gene association analysis in the samples of African Americans and Hispanic Americans as described below. Also, using the genome-wide identity-by-state (IBS) estimated with PLINK, we performed multidimensional scaling analysis on the matrix of IBS. Distributions of samples on the first to fourth dimensions were used to assess clustering and diversity within and between the groups.

### Global and local ancestries

To evaluate potential discrepancies between global and local population structures or ancestries in the samples we use the method of squared coefficients of canonical correlation 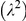 as described by Qinet al [34]. Briefly, let *N* denote the sample size, 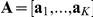 denote the 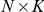 matrix consisting of the first *K* global principal components (PCs), and 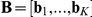 denote the 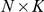 matrix consisting of the first *K* local PCs in a local window. The coefficient of multiple-determination 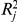 for 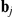 and 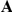 is the 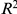 in the linear regression of 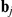 on 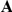. The *j^th^* largest squared coefficient of canonical correlation 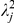 between 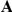 and 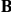 is the *j^th^* largest coefficient of determination between any linear combination of 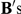 columns and any linear combination of 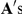 columns. The local PCs were computed from the local 20 Mb-window defined on each autosome. Squared coefficient was computed as the square of the largest canonical correlation between the first 10 local PCs of each local 20 Mb-window and the first 10 global PCs in each population sample. For this evaluation, we restricted analysis to the three Biobank samples (ANY, ENY and HNY) and three external comparison samples (AMW, BRZ and HapMap CEU).

### Structure analysis

Structure analysis was performed separately for the two marker sets using the software STRUCTURE [35], [36]. STRUCTURE applies a Bayesian model-based clustering algorithm to assign subjects into pre-assumed *K* ancestral populations each of which is characterized by a set of allele frequencies at each SNP. Based on their allele frequency profiles for the loci and under the assumption that loci are at HWE and linkage equilibrium within each population (racial group), the subjects are then probabilistically assigned to populations, or jointly to two or more populations if their genotypes indicated recent gene flow. For each of the two marker sets, analysis was run under an assumed number of ancestral populations ranging from *K* = 2 to *K* = 7. Analysis parameters included admixture model, correlated allele frequencies among populations, estimation of separate alpha for each population, and burn-in period of 20,000 iterations followed by 10,000 Markov chain Monte Carlo replications. Graphical displays of results of population structure were produced using the program DISTRUCT [35].

### Linkage disequilibrium and haplotype analysis

To compare LD structure and organization of haplotypes harboring published disease loci or loci that alter drug metabolism we carried out analyses of obesity-related (viz, fat mass and obesity associated (*FTO*) and melanocortin 4 receptor (*MC4R*)), and pharmacogenomic variants (viz, solute carrier organic anion transporter family, member 1B1 (*SLCO1B1*) and cytochrome P450, family 4, subfamily F, polypeptide 2 (*CYP4F2*)). This analysis was restricted to the Biobank sample. The software Haploview [37] was used to compute estimates of pair-wise LD by the standard D-prime method [38] and haplotype blocks defined by the confidence interval [39] using the standard parameter setting. All available SNPs in each gene were included in the analysis but comparisons between groups were restricted to haplotypes and LD blocks bearing or flanking the selected published disease or pharmacogenomic variants.

### Candidate gene association analysis for body mass index (BMI)

We carried out single SNP test for association between BMI and SNPs in *FTO* and *MC4R* genes. For each racial group, BMI was log-transformed to approximate trait normality. The residuals controlling for age and sex were standardized and used in association analysis with the SNPs. Only an additive genetic model was tested. The association analysis for the African-American and Hispanic samples was adjusted for population structure by inclusion of principal components as covariates.

## Results

The primary study sample was drawn from the 7,266 consented adult patients enrolled in Mount Sinai Medical Center Biobank from September 2007 to December 2009. Although the institution serves a diverse community in northern Manhattan, sampling was limited to self-identified African American (ANY), European American (ENY) and Hispanic (HNY) participants. Characteristics of the 977 individuals included in these analyses are presented in Table 1, including the frequencies of a set of common chronic conditions (asthma, CKD, diabetes and morbid obesity). All population structure analyses additionally included the ASW, MEX, YOR, AMW and BRZ samples (see Methods). The combined sample size including all population groups was 1770 and 2 separate pruned genotype marker sets with 100,133 and 28,732 SNPs were chosen, as described. Because the results from both marker sets were broadly similar, only results from the larger marker set are presented here.

**Table 1:** Characteristics of subjects.

	African Americans	European Americans	Hispanic Americans	All
N (% females)	324 (50.31)	326 (41.10)	327 (49.54)	977 (46.98)
Age (years)	52.69±13.77	49.55±14.39	56.11±13.72	52.79±14.20
Diagnosis (#without)	217 (107)	88 (238)	217 (110)	522 (455)
Diabetes	132	41	147	320
CKD	56	10	38	104
Obesity	90	38	74	202
Asthma	83	35	92	210

*Mean ± SD*.

### Multidimensional scaling of identity by state pair-wise distances

**Figure 1:**
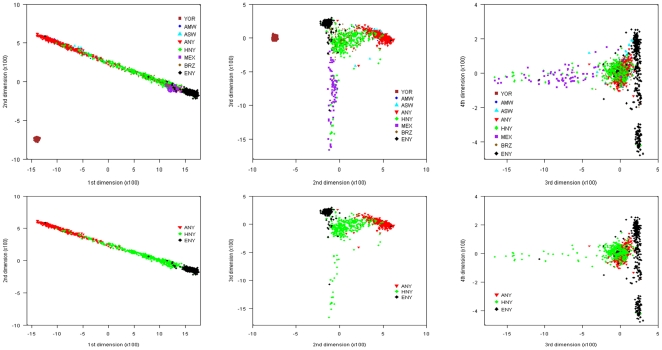
Multidimensional scaling plots for all samples (TOP) and only biobank samples (BOTTOM). Plots of subjects in the 1^st^ and 2^nd^ dimensions (left column), 2^nd^ and 3^rd^ dimensions (middle column), and 3^rd^ and 4^th^ dimensions right column). Abbreviation for samples: African American biobank sample (ANY); European American biobank sample (ENY); Hispanic American biobank sample (HNY); African ancestry in Southwest USA (ASW); Mexican ancestry in Los Angeles, California (MEX); Yoruba from Nigeria (YOR); African American from Maywood, Illinois (AMW); Brazilians from Brazil (BRZ).

To assess the within- and between-group clustering and diversity we performed multidimensional scaling analysis on the matrix of estimated IBS pair-wise distances and extracted the first four dimensions. Plots of the samples in the 1^st^ vs. 2^nd^, 2^nd^ vs. 3^rd^, and 3^rd^ vs. 4^th^ dimensions are presented in Figure 1; the upper panel describes all available population samples, including those from Nigeria and Brazil, while the lower panel is restricted to the 3 groups of patients from New York. Based on the 1^st^ and 2^nd^ dimension, the YOR sample formed a distinct non-overlapping cluster separated from all other samples, while the ENY sample also formed a small cluster. However, as best seen with extraction of the 2^nd^ dimension (middle panel, upper row), the groups designated as MEX, BRZ and HNY showed much greater dispersion, while partially overlapping with the ANY and ENY samples. This pattern is clearly consistent with recent gene flow from geographically distant populations among the Latin American groups. As anticipated, the three Biobank samples (ANY, ENY and HNY) tended to cluster with samples from similar reference population groups (i.e., ANY with AMW and ASW; and HYN with both the BRZ and MEX samples). When restricted to the Biobank samples, the HNY sample can be observed to cluster between the ANY and ENY samples and exhibited high within-group diversity which resulted in the observed dispersion (Figure 1).

### Global and local ancestry

**Figure 2:**
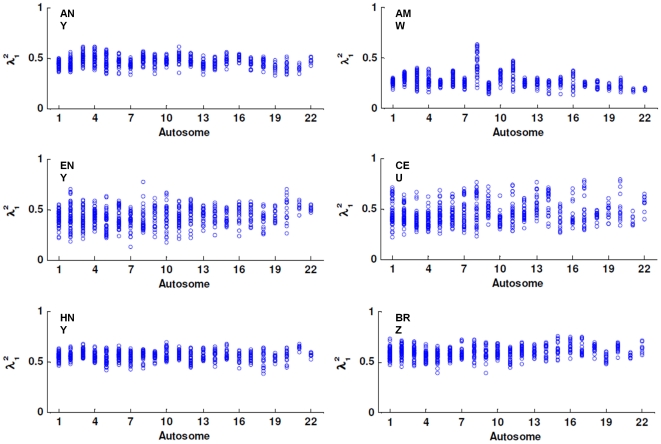
Canonical correlation based on window-wide local PCs for biobank samples and selected external samples. Each circle represents the squared coefficient of the largest canonical correlation between the first 10 local PCs of a local 20 Mb-window and the first 10 global PCs. Abbreviation for samples: African American biobank sample (ANY); European American biobank sample (ENY); Hispanic American biobank sample (HNY); African American from Maywood, Illinois (AMW); CEPH (Utah residents with ancestry from northern and western Europe (CEU); Brazilians from Brazil (BRZ).

The squared canonical correlation coefficients for the evaluation of discrepancies between global and local ancestry for each of the Biobank samples and corresponding external comparison samples are presented in Figure 2. In each sample, the squared coefficient is the largest canonical correlation between the first 10 local PCs of a 20 Mb-window and the first 10 global PCs. Criteria for choice of top 10 PCs and window size for local ancestry evaluation are as described in our previous study [40]. In each sample, we observed variation in the distributions of the squared canonical correlation coefficients from one autosome to the other, showing that ancestry or population structure is not uniform across the genome. It is expected that local genomic regions could be subject to varying forms of population structure as a result of natural selection, demographic history differences and local random fluctuations of admixture, among others [34]. These differential distributions of local population structures are also, to some extent, evident between samples of similar ancestry and this could have been amplified by sampling variation or genotype batch effects associated with the genotype calling algorithms of different platforms [41], [42], [43], [44], [45], [46], [47].

### Population structure

**Figure 3:**
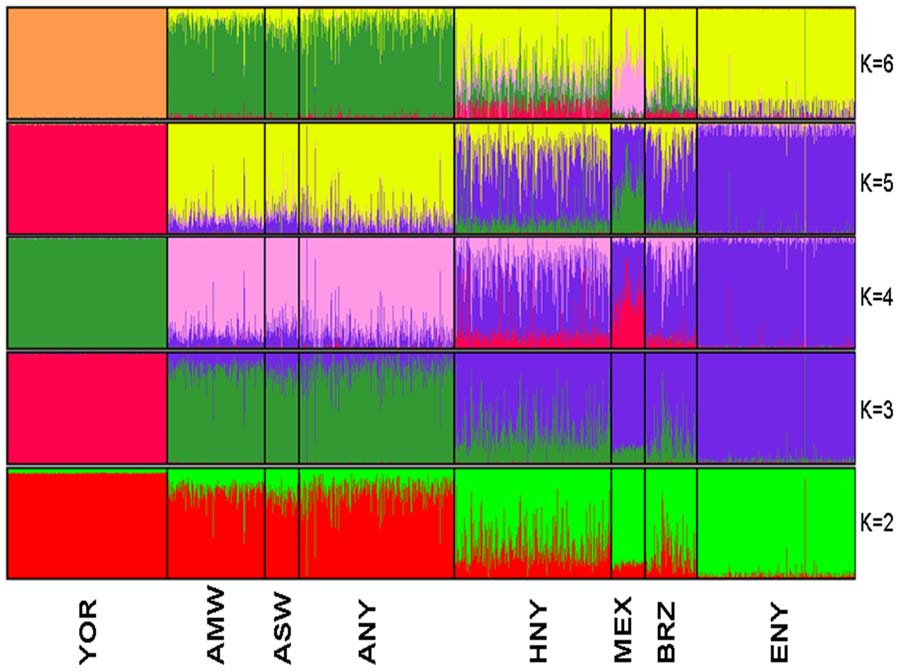
Population structure results for ancestral populations *K* = 2 to *K* = 6. Each subject is represented by a thin vertical line colored in proportion to their estimated ancestry within each cluster. The colors represent the proportion of inferred ancestry from each of the ancestral populations within each specific K value. Abbreviation for samples: African American biobank sample (ANY); European American biobank sample (ENY); Hispanic American biobank sample (HNY); African ancestry in Southwest USA (ASW); Mexican ancestry in Los Angeles, California (MEX) ; Yoruba from Nigeria (YOR); African American from Maywood, Illinois (AMW); Brazilians from Brazil (BRZ).

We carried out further analysis of population diversity using the software STRUCTURE (Figure 3). Both the YOR and ENY samples were assigned to separate populations. Evidence of recent gene flow was can be seen as shared colors that correspond to the proportions from each ancestral group. The ANY (and also AMW and ASW) sample exhibited a higher proportion of ancestry from Africa than from Europe. On the other hand, the HNY sample on average exhibited higher proportion of ancestry from Europe than from Africa. We likewise observed more within-population variation of individual ancestral proportions in the HNY sample. As *K* increased from 2, each sample with the exception of the YOR showed varied levels of admixture of the assumed ancestral populations. Closer inspection indicated that the admixture seen in ANY and HNY best supports the presence of 2 and 3 ancestral populations, respectively. In addition, evaluation of model estimates for the different values of *K* indicated a better fit for *K* = 2 and K = 3 for the ANY and HNY samples, respectively.

**Figure 4:**
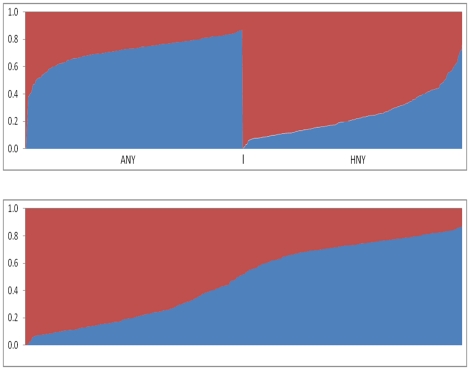
Population structure results for *K* = 2 ancestral populations sorted by ancestry proportions for African American biobank sample (ANY) and Hispanic American biobank sample (HNY) (Top) and pooled sample of both ANY and HNY (Bottom).

While clear differences exist in terms of the proportion of continental ancestry between the Hispanics and African Americans in this sample, this result is in part an artifact of the use of group labels. In fact, ancestral heritage at the individual level among persons from these two groups is best represented as a continuum (Figure 4).

### LD structure and haplotypes harboring published disease variants

To assess within–population group LD structure and the distributions of haplotypes harboring known disease variants, we selected published obesity and pharmacogenomic variants that were genotyped in our Biobank sample of African Americans, European Americans and Hispanic Americans. For obesity variants, we selected 7 *FTO* SNPs (rs1421085, rs1121980, rs8057044, rs8050136, rs9939609, rs9941349 and rs9930506) and 2 *MC4R* SNPs (rs17782313 and rs12970134). For pharmacogenomic variants, we selected rs4149056 and rs11045819 in the *SLCO1B1* gene and rs2108622 in the *CYP4F2* gene. The *SLCO1B1* variants affect response to 3-hydroxy-3-methylglutaryl-coenzyme A (statins) [48], [49], [50] and the CYP4F2 variant affects response to warfarin [1], [51]. We included all available SNP genotypes in these genes in the determination of the LD blocks and hyplotypes within each group. Here, we present results from same region across groups for the purpose of comparison.

**Figure 5:**
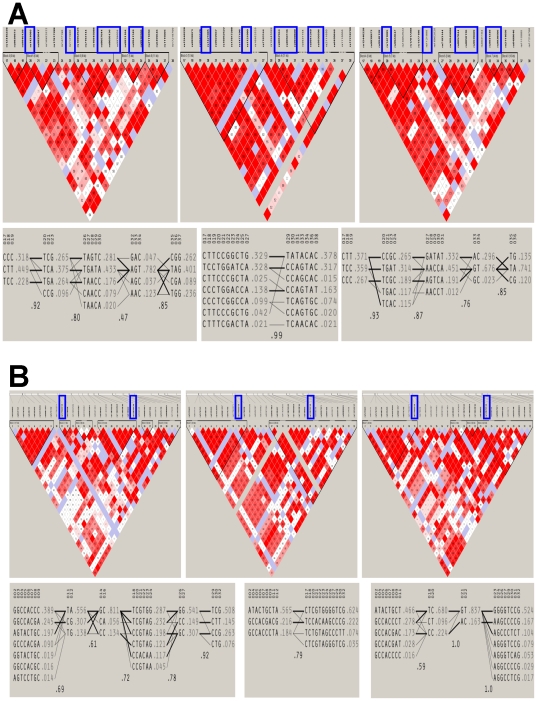
**a:** Linkage disequilibrium structure (Top) and organization of haplotypes harboring published obesity variants (rsIDs indicated with blue boxes) in **FTO** gene in biobank sample of African Americans (left column), European Americans (middle column), and Hispanic Americans (right column). **b:** Linkage disequilibrium structure (Top) and organization of haplotypes harboring published obesity variants (rsIDs indicated with blue boxes) in **MC4R** gene in biobank sample of African Americans (left column), European Americans (middle column), and Hispanic Americans (right column).

Results of the LD structure and distribution of haplotypes bearing *FTO* and *MC4R* variants are shown in Figures 5a and 5b. Substantial differences were observed between groups in the number and length of LD blocks in the targeted regions. For instance, in the *FTO* region there were 5 LD blocks in the African American sample, 2 LD blocks in the European Americans, and 4 in the Hispanics (Figure 5a). Similarly in the *MC4R* region, there were 6, 2 and 4 LD blocks in the African American, European American and Hispanic American samples, respectively (Figure 5b). The observed strength of LD within blocks varied widely between groups just as the number of haplotypes in every block also varied between the three groups. As a result of the variation in the number and length of the LD blocks, haplotypes bearing or flanking the susceptibility variants were clearly not always comparable.

**Figure 6:**
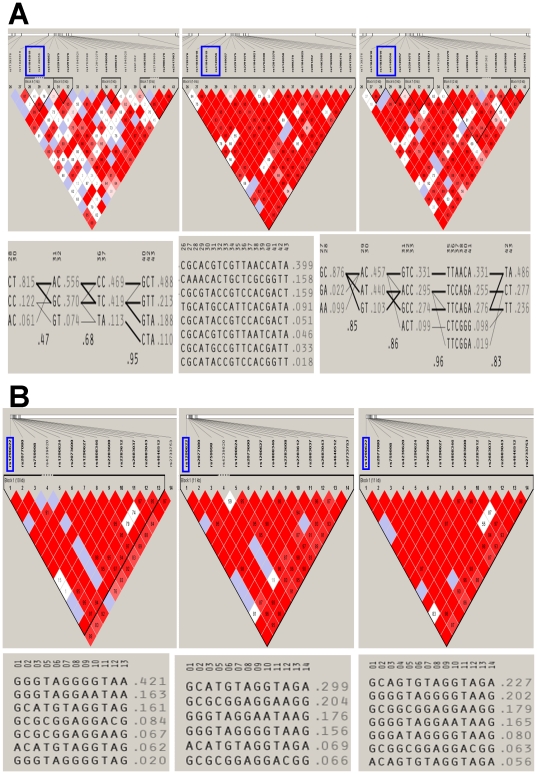
**a:** Linkage disequilibrium structure (Top) and organization of haplotypes harboring published pharmacogenomic variants (rsIDs indicated with blue boxes) in **SLCO1B1** gene in biobank sample of African Americans (left column), European Americans (middle column), and Hispanic Americans (right column). **b:** Linkage disequilibrium structure (Top) and organization of haplotypes harboring published pharmacogenomic variants (rsIDs indicated with blue boxes) in **CYP4F2** gene in biobank sample of African Americans (left column), European Americans (middle column), and Hispanic Americans (right column).

Similar discordance between LD structures and haplotype distributions was observed for the pharmacogenomic variants. In the *SLCO1B1* gene, there were 4, 1 and 5 LD blocks with widely different number of haplotypes in the African American, European American and Hispanic American samples, respectively (Figure 6a). It is important to note that the length of the single block in the European-American sample spanned the same region in which multiple blocks were found in the other two groups. As a consequence few SNPs tagging this haplotype would be observed in the African-American and Hispanic samples making it difficult to validate associations originally discovered in European Americans. Results for the *CYP4F2* variants are shown in Figure 6b. Again, the racial groups differ from each other in terms of length of LD block and number of haplotypes.

### Candidate gene association

**Figure 7:**
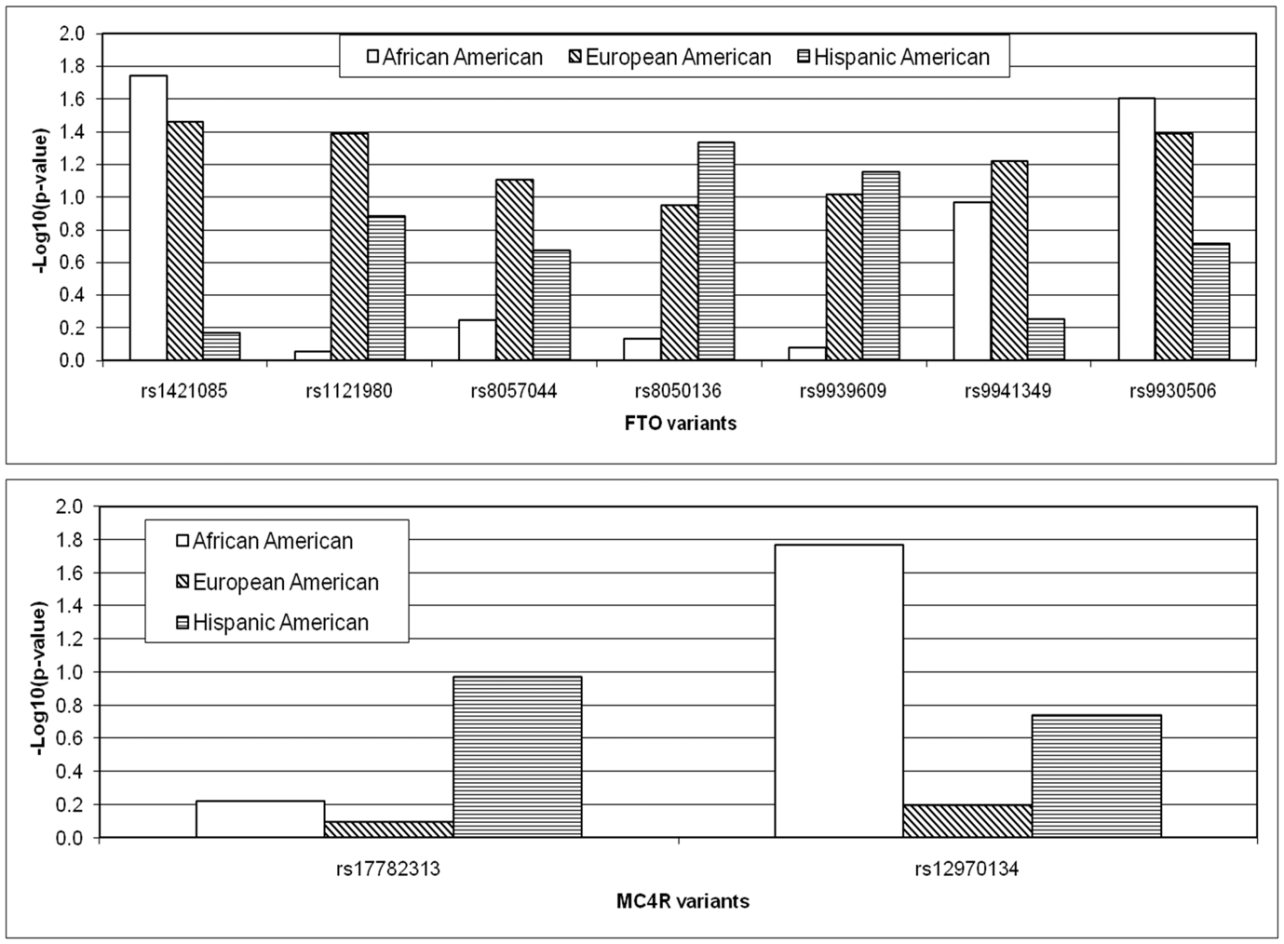
BMI associations with published obesity variants in FTO (Top) and MC4R (Bottom) genes in the three biobank samples.

As an illustrative example, results of comparative association analysis of the association between BMI and haplotypes tagged by published SNPs in the *FTO* and *MC4R* loci are presented in Figure 7. Although these loci have provided the strongest signals for adioposity, the effect sizes are admittedly modest (≤∼1.3 for BMI>30) and therefore large sample sizes are required to obtain stable results. Nonetheless, it is clear that among European Americans the association is consistent across this region, while highly variable for African Americans and Hispanics. The *MC4R* region includes fewer haplotypes and the underlying effect size is smaller than for *FTO*, and is therefore less informative. The pattern in Figure 7, however, suggests that the association with BMI is less well captured by the common haplotypes than for the other ethnic groups.

## Discussion

Genomic technology has rapidly transformed the character of biomedical research, however the translational impact of this new science for clinical medicine remains undefined. Some investigators have suggested that the current state of knowledge justifies sequencing individual patients' genomes and placing this information in the electronic medical record, although others hold that the current utility of genome sequence data is still far too limited, while the storage burden and interpretation are unsupportable, and this state of affairs is unlikely to change in the foreseeable future [2], [13], [52], [53]. Clearly more incremental steps will be required in order to adapt genomic science for clinical purposes and evaluate its contribution to patient outcomes. One of the most immediate challenges is the need to quantify and properly account for the genetic diversity present in clinical populations. Formal research studies have virtually always stratified on a conventional descriptor of population structure and diversity is subsequently assessed within and between groups [18], [20], [23], [54]. While this approach has been useful in describing global patterns, others have argued that it narrowly “packages” our view of human variation [55]. Even more problematic in the clinical arena, racial/ethnic labels can vary widely by geographic location and time (eg, Hispanic and Asian) and cosmopolitan cities now include many individuals whose genetic heritage is drawn from multiple continental origins.

The analyses presented here attempt to capture the pattern of genetic diversity in patients seen at a major medical center in New York City. Consistent with previous studies of population genetics, broad ancestral clustering is apparent in the 3 patient groups [18], [23], [54]. At the same time, wide divergence is seen in the Hispanic samples. Using the traditional perspective that “packages” geographic populations, these sub-clusters roughly represent Mexican Americans, persons of Caribbean origin (who would overlap with Brazilians), and a third group that clusters with African Americans [54]. Conversely, if one dispenses with the conventional labels and relies solely on genotype the Hispanics in the NY sample can be appropriately merged with the African Americans (Figure 3). These data forcefully underscore the diminishing relevance of the descriptors currently used for the two principle minority groups in the US.

A complementary layer of complexity is demonstrated by the illustrative examples based on genetic variants associated with common traits and drug response. As is well recognized, both the spectrum of allele frequencies for “causal” mutations and proxy haplotypes can vary widely across population groups [15]. As this phenomenon has become better appreciated, the relevance of “diagnosis by proxy” using race/ethnicity to predict genotype, has dissipated [56]. Since the vast majority of current findings from GWAs are based on SNPs that tag the relevant haplotype, until the “causal mutation” is known for these findings it may not be possible to transfer this information from the original study population, virtually always of European ancestry [15]. The relevance of cross-population haplotype diversity was perhaps most clearly demonstrated in the analyses that defined the causal mutation at the *SORT1* locus [4]. A variant at this locus confers the largest effect on LDL-cholesterol of any known common allele [4]. While a broad haplotype carrying numerous SNPs was captured on GWAs in a European-origin sample, the apparent causal mutation was isolated to a smaller haplotype present in persons of African descent [4]. It must be assumed, therefore, that proxy haplotypes cannot be used for individual-level patient analyses.

While the sample included in this study was restricted to a single hospital the inferences are generalizable to most urban centers in the US. The combination of rapid changes in migration away from Europe and the realization that very few genetic variants are sufficiently differentiated even between historically unrelated populations mean that clinical decisions about genotypic effects will require very detailed knowledge of the locus in question, and analyses of individual patients. In a sense, therefore, much of the research emphasizing continental origin and ancestry stands in contradiction to the clinical imperative and a shift away from a paradigm that is founded on racial/ethnic categories will be required. We would suggest that this shift in perspective will be one of many required before genomic science can be fully adapted to use in the clinical arena.

This report has limitations which must be recognized. The racial/ethnic background of patient populations will, of course, vary widely across the US the specific composition observed in New York may not be observed. We did not have access to appropriate data on Native Americans that might have helped define genetic ancestry of migrants from Mexico or other parts of Central and South America. Recent analyses by Bryc *et al* demonstrate the heterogeneity with Hispanics sub-groups and our data are consistent with their results, albeit weighted toward persons from the Caribbean [18], [23], [54]. We also recognize that clinical testing must take place in approved laboratories and will be restricted to genotypes that have been widely validated as relevant for patient outcomes therefore data from GWAs must be filtered extensively before application in any group. This process of defining the functional variant will by itself de-emphasize the broad framework of race/ethnicity.

In conclusion, based on a consecutive series of patients from an urban medical center in New York City we demonstrate that a spectrum of mixed ancestry is emerging in the largest US minority groups. While consistent with previous descriptive studies, when viewed from the clinical perspective this evidence invites a re-evaluation of the relevance of racial/ethnic labels. In combination with evidence of locus heterogeneity within and between populations, this picture of extensive gene flow lends credence to the argument that the transfer of historical population labels which reflect language and other social categories onto patient samples will in many cases be unwarranted.
